# Open Dialogue as a Human Rights-Aligned Approach

**DOI:** 10.3389/fpsyt.2019.00387

**Published:** 2019-05-31

**Authors:** Sebastian von Peter, Volkmar Aderhold, Lauren Cubellis, Tomi Bergström, Peter Stastny, Jaakko Seikkula, Dainius Puras

**Affiliations:** ^1^Department of Psychiatry and Psychotherapy, Medical University Brandenburg, Neuruppin, Germany; ^2^Department of Psychiatry and Psychotherapy, Medical University Greifswald, Greifswald, Germany; ^3^Department of Anthropology, Washington University St. Louis, St. Louis, MO, United States; ^4^Department of Education and Psychology, University of Jyväskylä, Jyväskylä, Finland; ^5^Community Access, New York, NY, United States; ^6^Department of Psychiatry and Psychology, Vilnius University, Vilnius, Lithuania

**Keywords:** mental health, crisis, promotion, violations, universal

## Abstract

Throughout the last 20 years, the human rights perspective has increasingly developed into a paradigm against which to appraise and evaluate mental health care. This article investigates to what extent the Finnish open dialogue (OD) approach both aligns with human rights and may be qualified to strengthen compliance with human rights perspectives in global mental health care. Being a conceptual paper, the structural and therapeutic principles of OD are theoretically discussed against the background of human rights, as framed by the Universal Declaration of Human Rights, the UN Convention on the Rights of People with Disabilities, and the two recent annual reports of the Human Rights Council. It is shown that OD aligns well with discourses on human rights, being a largely non-institutional and non-medicalizing approach that both depends on and fosters local and context-bound forms of knowledge and practice. Its fundamental network perspective facilitates a contextual and relational understanding of mental well-being, as postulated by contemporary human rights approaches. OD opens the space for anyone to speak (out), for mutual respect and equality, for autonomy, and to address power differentials, making it well suited to preventing coercion and other forms of human rights violation. It is concluded that OD can be understood as a human rights-aligned approach.

## Introduction

The human rights perspective on mental health has a long tradition (1). It has gained importance largely since the publication of the WHO’s guidelines in 1996 (2), increasingly developing into a central paradigm against which to appraise and evaluate mental health care worldwide. Today, human rights concerns are at the foreground of international considerations (3), paired with the principle of scientific evidence in guiding global mental health care and promotion (4).

In his latest report (5), the UN Special Rapporteur for the Right of Everyone to the Enjoyment of the Highest Attainable Standard of Physical and Mental Health carries this emphasis on human rights further: Introducing the principle of “human rights first” (p. 1), he eschews the traditional supremacy of scientific evidence over other rationales for the promotion and implementation of health care interventions. According to his argument, justifying an intervention’s significance should require not only a consideration of the existing evidence but more importantly its potential to align with and strengthen human rights.

The UN Special Rapporteur’s report has evoked extensive debates (6, 7) and also criticism (8). These debates are of critical importance, as human rights in the context of mental health is a worldwide concern (9). Without appropriately responsive interventions, mental health problems may hold damaging potential for the persons concerned and for their social and cultural surroundings. Yet intervening in the name of mental health also may bring along the risk of human rights violation. This includes not only rather obviously transgressions, such as detainment, isolation, restraint, and medication without consent, but also more “silent” and hidden violations that may exclude, stigmatize, pathologize, depersonalize, or disempower people living with mental distress.

Various reports have confirmed the severity of human rights violations affecting people with mental health problems in almost all cultures, though there are variations in frequency, intensity, or severity (10, 11). As a response, various policies and regulations have been established in recent decades, differing from one country to another, and raising various concerns about global disparities. At the same time, around the world, there is a long history of injustice embedded in the mental health care sector, and this provokes the challenging question of how to change current practices, which have been routinized over centuries (10).

Thus, essential changes of the worldwide mental health care sector are needed, requiring more than a handful of trainings or a quick modification of practices (10). Instead, fundamentally new ways of understanding and responding to mental health problems must be established to better comply with human rights policies and regulations. To clarify this argument, a productive example is given *via* the analysis of the open dialogue (OD), an approach that will be understood in the following as a means by which to foster human rights in mental health care practices. OD is a set of network and community-centred techniques that originally served as early intervention practices to treat persons with psychotic experiences (12). Of the “twin principles” (4), OD meets the criteria of being scientifically well evidenced: Largely focusing on Scandinavian research fields, there is a set of quite robust cohort and descriptive studies that demonstrate its real-life effectiveness (12–15).

On the other hand, the compatibility of OD with the principles of human rights has not been demonstrated yet, prompting the following research questions: To what extent does OD as a well-defined approach aligns with human rights, in the sense of qualifying as a means by which to realize and protect human rights in mental health care practice around the globe? This question is intended to generate not only an OD-related analysis but also a more general discussion of how various principles of human rights may serve to substantiate the benefits of defined mental health interventions.

## Materials and Methods

When Jaakko Seikkula and his team in Finland developed the model known today as the OD approach, systematic changes were initiated on two different but equally important levels (16): First, a culture of dialogical communication between staff, patients, and relatives was established. Second, community-based, multi-disciplinary treatment teams were organized and deployed to offer primarily outpatient services. A low medication- and primarily psychotherapeutically orientated treatment approach, involving a processual understanding of psychiatric crisis and disabilities as meaningful reactions to a specific context (16), are key values of OD that have been further elaborated upon over time.

OD developed from the need-adapted treatment model (NATM), also conceived in Finland by Y. Alanen and his team since 1975 (17). NATM draws on the work of the Norwegian psychiatrist T. Anderson on reflecting processes and a social constructionist view of relationships (17). Starting with an individualist, psychodynamic model to treat people with psychotic experiences, NATM soon came to include a network approach, carrying out therapeutic activities flexibly and specialized to meet the changing needs of the persons concerned and their networks. OD can be perceived as a further refinement of NATM, introducing dialogic elements into these therapeutic practices.

OD is now practiced in various regions around the globe, i.e. in various parts of Scandinavia, Germany, the Netherlands, Austria, the UK, the USA, Australia, and even Japan. Instead of representing a clearly demarcated intervention, OD varies internationally in its adaption to local care systems and contingencies. Yet a set of circumscribed principles have been recently developed that are central to most implementation of OD practices (16): 7 structural principles delineate ways of (re-)structuring the treatment organization, and 12 therapeutic principles outline the specific approach towards the persons concerned and their network. These principles currently constitute the OD fidelity criteria. They will be discussed in depth within the Results section but are also listed in **Table 1**.

**Table 1:** The seven structural and 12 therapeutic principles of OD practices (16).

No.	Structural principles (sp)	Therapeutic principles (tp)
1.	Immediate help	Two (or more) therapists in the team meeting
2.	Social network perspective	Participation of family and network
3.	Flexibility and mobility	Using open-ended questions
4.	Responsibility	Responding to clients’ utterances
5.	Psychological continuity	Emphasizing the present moment
6.	Tolerance of uncertainty	Eliciting multiple viewpoints
7.	Dialogue	Use of a relational focus in the dialogue
8.		Responding in a matter-of-fact style and attentive to meanings
9.		Emphasizing the clients’ own words/stories, not symptoms
10.		Conversation among professionals in the treatment meetings
11.		Being transparent
12.		Tolerating uncertainty

Concepts of human rights are contested and heterogenous (18). Human rights are not absolute but rather must be balanced against other rights and competing public interests (10). Human rights certainly do not concern only the therapeutic situation but instead are bound up with various spheres and relationships, particularly those between the state and its citizens. Yet, in this article, only human rights that are relevant within the mental health service context are considered, shaping the various ways that these stakeholders relate and communicate with each other.

Therefore, this article uses various frameworks of human rights, referring to both the Universal Declaration of Human Rights (UDHR) (19) and the UN Convention on the Rights of People with Disabilities (CRPD) (20). Both declarations strongly rely on the principles of self-determination and providing the affected people with a voice, both of which are at the core of the radically need-adapted OD approach. In addition, this conceptual analysis draws on the two recent annual reports of the Human Rights Council (HRC) on mental health and human rights (21, 22), as these presently engage with the subject of human rights in global mental health care.

All cited references use various accounts and concepts to frame the human rights of people with mental health problems, such as individual autonomy, freedom of choice, non-discrimination, full and effective participation and inclusion in society, respect for difference and diversity, freedom of speech and expression, bodily integrity, freedom of arbitrary detention, and restraint and detainment. They also relate to other human rights such as the right to adequate housing, just and favourable conditions for work, equal recognition before the law, and freedom of various forms of deprivation. Although all these rights are of central concern for people with mental health problems, referring to all of them would extend the scope of the clinical situation. Yet it will be shown that OD as a practice may be a means to address these kinds of rights as well, an argument that is of great importance given the fact that these dimensions of lived experience strongly correlate to mental health and mental health problems (18).

Methodologically, this article is a conceptual paper. It discusses the conceptual framework of OD (the above-mentioned structural and therapeutic principles) against the background of human rights, as framed above. Thus, theoretical considerations will be at focus in the following, with the aim of investigating how closely the principles of OD correspond to those of human rights. At the same time, these considerations are broadly influenced by the authors’ day-to-day clinical practices and other forms of their professional or scientific experiences with OD. Thus, the article is infused with various forms of experiential knowledge, making it rather like a hybrid between theoretical and practical forms of knowing.

## Results

### Post-Psychiatric Understanding of Crises

OD employs a crisis perspective on mental health problems. According to the structural principle (sp) of immediacy (sp1—see **Table 1**), within the first 24 h of a crisis situation, help is offered *in situ* (sp3), i.e., outside of mental health care facilities and within the everyday environment of the person or networks of concern (sp2). Decisions about the location and timing of the first and subsequent network meetings are left to the clients themselves (sp3), and the treatment team is at their disposal (sp4), working to ensure availability and personal continuity (sp5).

These structural conditions indicate a fundamental shift away from what were traditionally institutional concerns, routines, and logics: Rather than turning to hospital-based or authoritative interventions, OD directs treatment towards a more patient-determined, open, and everyday life-related procedure. Correspondingly, dialogic practices, i.e., the therapeutic principles (tp), largely focus on meaning-making and an understanding of life-related problems (tp8). Central to this is the therapists’ openness towards the clients’ own accounts (tp6) of their experiences, and their subjective explanatory models (tp3). Professional diagnoses or clinical classifications are secondary, whereas the clients’ own language is prioritized during the network meetings (tp9).

Taken together, these principles demonstrate that OD is a largely non-institutional and non-medicalizing approach. Promoting the clients’ potential for self-exploration, self-explanation, and self-determination, OD turns away from an institutionally driven agenda. Focusing on human response and communication, the network is supported in making sense of crisis as a *life event* rather than as a medical condition. Accordingly, pragmatic and life-related, instead of medically informed or manualized solutions, are sought, thereby fostering possibilities related to and embedded in everyday life, human relationships, and mutual understanding.

Such an approach exists in agreement with a post-psychiatric (23) perception of mental health care epistemology and practice, which prioritizes basic human values and everyday relationships, context-bound understandings, and local belief systems over symptoms and clinical diagnostics. This shift resonates well with a human rights approach for a number of reasons: First, it corresponds to the framework of human dignity and the value of the subject, as outlined within the UDHR (Art. 1). Instead of objectifying a person by applying a diagnostic label, he or she is appreciated as a full human being, capable of meaning-making, understanding, and acting upon themselves and the world around them. Endowed with reason and conscience, human nature is perceived to be inherently bound to subjective accounts and understandings, making communication *the* central response in moments of crisis.

Second, such an approach is compatible with the CRPD’s relational model of disability, which conceptualizes a complex interplay between individual and environmental factors as both cause and perpetuator of (also psychosocial) disabilities (21, 25 Art. 1). The CRPD uses a relational notion of disability, reciprocally connecting individual deficits to contextual constraints: Neither an impairment nor an environment alone is perceived to be disabling *per se*, but only the combination, or better, the discrepancy of both, impeding a person’s participation in society. Thus, both the OD principles and the CRPD foster an understanding of crisis that is deeply embedded within life conditions, contrasting to a more traditional medical perspective that perceives a disease to be an inherent trait of a person prevailing across situations (24).

And finally, such an approach relates to both of the HRC reports, arguing that the use of primarily the medical model may lead to further stigmatization and exclusion of people with mental distress (21). Within these reports, the biomedical model of mental health is held accountable for fostering an “increasing gulf of exclusion” (21) between persons with psychosocial disabilities and the communities in which they live. The medical framing of mental health is characterized as inducing social distance and a fundamental othering of the persons concerned (22). In contrast, a post-psychiatric understanding conceptualizes mental health problems to be of a universal kind—everybody may be affected at any time, depending on their life situation and state of being—making a mental crisis an essential part of human nature.

### Strengthening Networks and Promoting Social Cohesion

A major structural principle of OD concerns the provision of treatment *within* the interactional and broader social network of the person of concern (sp2). Fundamental to dialogic practices is the systematic integration of the client’s family and his or her close network during the network meetings (tp2). To facilitate this, everyone should be listened to and given the possibility of speaking, not just the person identified as experiencing problems or symptoms (tp6). Relational questions and other forms of dialogic inquiry (tp7) are employed to develop a shared language and shared meaning system for talking about the crisis (sp7). The aim is to understand the crisis as a natural reaction to a difficult situation (tp8).

Certainly, there are other therapeutic approaches that use a social network perspective, especially systemic forms of psychotherapy, to which OD owes many of its elements. Yet, in OD, the network perspective is *the* central component, the lynchpin that governs all its structural and therapeutic principles. Thus, the network meetings are implemented, whenever possible, as the first step of treatment, even in acute states of psychosis. Furthermore, the network approach of OD is characterized by its fundamental openness towards possible outcomes—what constitutes solutions to the crisis are not known in advance. Finally, other forms of systemic therapies usually are not recommended for people with so-called severe mental illness, whereas OD has been especially developed for this group of persons. In contrast to other systemic approaches, therefore, the social network perspective is the central organizing element of OD and is available to people and networks with all sorts of crisis experiences, facilitating open engagement with the problems at stake.

The network meetings may involve actors from various fields: family members and other kin, neighbors, and friends, and also more formal actors such as school teachers, social workers, employers, and (traditional) healers. Dialogue may be promoted among all these actors, building networks across various life worlds and levels of society. Thus, OD might also be understood as a means of community enhancement. Following a socio(-ecological) paradigm of mental health and mental health care, it stands to promote social cohesion. Emphasizing relationality and dialogue, OD strengthens interconnectedness and mutual trust and therefore may promote not only the health of the individual but also the collective well-being of a community or social system.

This social network perspective prevents the exclusion of network members, as often occurs when traditional, more individualistic and medically orientated treatment logics are used. Thus, it corresponds to an inclusionary approach, as it has been outlined in the CRPD: Everyone has the right to full and effective participation and inclusion in society (Art. 3), services must be accessible to all (Art. 9), and everybody has the right to receive and impart information and ideas on an equal basis with others and through all forms of communication of their choice (Art. 21). In this sense, the central organizing principle of the social network perspective and the open and non-compulsory ways of communicating within the network meetings together enable all relevant actors to freely participate and engage in the processes of understanding and finding solutions for the problems at stake.

Further, the social network perspective corresponds with the CRPD’s approach to disabilities: If disability depends largely on context (21, Art. 1), then context must be addressed in the corresponding solutions (24). Both the CRPD and OD employ a relational approach to find solutions for the problems at stake. In both cases, context is most important in both understanding a problem and finding a solution. Underlying this understanding of context dependence is, in both cases, a particular notion of personhood: to be in the world is to be *related*, to be *engaged*, to be *embedded*—social networks are central to the experience of being human and the origin of both challenges and resources in navigating moments of crisis.

This emphasis on context is also important in both the HRC annual reports, though transcending the impact of the immediate social network: Both reports stress the importance of social, economic, and political determinants of mental health problems, such as various forms of discrimination and stigmatization; unequal access to housing, work, and other resources; and other forms of lifeworld-related disparities (21, 22). In this context, a social network perspective is well suited to direct attention to this plethora of interconnected variables. OD as an approach may be used to explicate how these determinants and inequalities impact mental health and thus may serve to bridge both micro-levels and macro-levels of society.

### Restoring Dignity and Fostering Equality

In OD meetings, transparency is of the highest value (tp11): All information is shared, every decision is discussed within the network meetings, and the therapists openly reflect on their own thoughts, making them available for discussion (tp10). Furthermore, in network meetings, all voices are to be heard (tp6), no voice should be favored or dominant (sp7), and every person should be treated with utmost equality (tp2). Each person is invited to speak out, using their own words and stories (tp9), even psychotic experiences; and the therapists’ primary task is to be open (tp3) and largely responsive, rather than instructive or interpretive (tp4).

In this sense, OD techniques can help restore human dignity: Dialogically, a common language is developed through the meeting to support the network in the search for words to name the previously unspeakable (pt9). Thereby, any expert voices, medical expertise included, is converted into part of the polyphonic exchange, rather than providing a dominant or authoritative frame. Within this dialogic process, there is no right or wrong; OD does not strive for consensus but rather for the generative juxtaposition and creative exchange of multiple viewpoints (tp6). All actors are respected as full human beings and are encouraged to speak from their own positionality (pt12). Recognizing diversity and the worth of every single voice is essential to dialogic practices.

Additionally, OD is very explicitly a non-hierarchical approach. Horizontal forms of multi-professional collaboration are necessary to create space for dialogical communication (tp1). Power dynamics between providers and users of services, as well as within the network, should be addressed openly and modified accordingly (tp11). Clients and networks are encouraged to (re-)claim agency in making their own choices about their health and treatment procedures (tp3), demonstrating the emancipatory and empowering potential of dialogic practices.

Allowing a person to find and express the right words for the previously unspeakable makes him or her a human being, with a voice of consequence that is heard and responded to. Correspondingly, the preamble and Art. 19 of the UDHR as well as Art. 21 of the CRPD reinforce freedom of speech, belief, and expression. The persons concerned have a right to speak out and to express themselves, as do all human beings, in whatever condition and situation they may find themselves. Each of us is born free and equal in dignity and should enjoy the same rights.

Furthermore, the OD principles of respecting diversity and multivocality during network meetings resonate well with the CRPD aim towards inclusionary practices and participation (21, Art. 3). OD practices do not strive for consensus; instead, they aim at the non-judgmental juxtaposition of various perspectives. As human beings are distinct, these differences must be accepted and allowed for and understood to be part of humanity’s diversity of experiences.

Finally, in both codes, and in agreement with OD practices, autonomy and self-determination are central values (21, Preamble; 20, Preamble). The CRPD (Art. 3) recognizes the importance for people with disabilities of their individual autonomy and independence, including the freedom to make their own choices. Persons with disabilities should have the opportunity to be actively involved in decision-making processes, including those directly concerning them. In the same way, the HRC annual reports advise treatment systems that radically redistribute power and hold both equality and freedom of choice in high esteem (21, 22). This corresponds to the ODs radically person-centred approach that supports the ability of all persons concerned to reclaim agency and decision-making power.

## Discussion

This article employs a highly idealized account of OD. OD in practice surely falls short of this idealization, itself subject to the complexities of context, funding, and implementation. But the same is true for the implementation of any intervention. A further limitation is the authors’ bias in largely focusing on commonalities, instead of distinctions, between the principles of OD and human rights; yet discrepancies were difficult to detect, maybe also due to the authors’ enthusiasm for OD. Third, OD largely shares significant similarities with a variety of other concepts or approaches, such as the NATM, Soteria [a non-clinical, relation-based but rather stationary approach to attend people with psychotic experiences (25)], and various systemic approaches. While our discussion was necessarily narrowed to focus on OD, it is important to recognize the multiplicity of relations and influences that shape its history and current practices. At the same time, this article may serve as a starting point to consider also the compatibility of many other approaches with human rights values and principles.

Despite these limitations, this conceptual paper aimed to demonstrate that the distinctive principles of OD are well aligned with contemporary discourses on human rights. OD is a largely non-institutional and non-medicalizing approach, which both depends on and fosters local and context-bound forms of knowledge and practice. Its fundamental network perspective facilitates a contextual and relational understanding of mental well-being, as it is postulated by contemporary human rights approaches. OD opens the space for anyone to speak (out), for mutual respect and equality, for autonomy, and for both recognizing and addressing power differentials.

In this sense, we argue that OD can be understood as a human rights-aligned approach. Yet there is a major limitation to this argument, as the mentioned studies on OD did not investigate its impact on any forms of coercion, such as rates of forced or non-consensual treatment, detainment, isolation, and other forms of restraints, with exception to one study that demonstrated a reduction of involuntary admissions in the regions that practiced OD and compared with Finland (26). Currently, there is an extensive and controversial debate on coercion and psychiatric practices, particularly on how to understand Art. 14–16 (rights to liberty, freedom of inhumane treatment, and punishment and freedom from exploitation, violence, and abuse) of the CRPD in relation to mental health care (27, 28). There is no easy solution to these questions. At the same time, answers must be at the center of any human rights-aligned approach.

Thus, future studies in OD must address its impact on various forms of coercive measures. This is even more desirable as the principles of OD seem to be well suited to prevent coercive practices and other forms of restraint. Fostering safe spaces of mutual understanding and engagement, the basic values of OD, relate to consensus, participation, autonomy, dialogue, and communication. OD is practiced primarily in non-institutional settings, making coercion less likely. Taking up a social network perspective facilitates a shared form of “risk management” by involving a network of mutual support and distributed responsibility. As a result, OD as an approach is mentioned in various reports as a promising practice to reduce coercion (5, 6). Yet solid evidence for this argument is still pending.

Further, and to conclude, OD as a treatment model is adaptable to various cultural and structural conditions: First, as it does not maintain a preference for any specific explanatory model, OD can function as a non-essentializing, culturally sensitive approach in a variety of environments and ecologies. Furthermore, framing treatment difficulties as social rather than medical concerns makes OD less dependent on highly educated psychiatrists or other professionals. Instead, it can involve lay (and easily peer-) workers that have been adequately trained and may be employed in the primary health care sector (29). This flexibility, and the fact that OD processes are largely community-based and consume rather moderate resources, suggest that OD could be a universally applicable approach.

It remains to be seen whether OD might serve as a potential catalyst in the promotion of human rights, not only in the field of mental health, but also in more general ways and around the globe. Worldwide, human rights principles are subject to many challenges. Autocratic regimes and military conflicts place a heavy burden on the daily lives of many people. Conversely, there is a considerable lack of feasible and locally applicable instruments for promoting peace and social cohesion. In this sense, the OD approach may be understood not only as a means for advancing mental well-being but also a means to foster human rights in more general ways. Although this expansive imagining of OD may appear idealistic at first sight, the authors believe that engaging with human rights discourse can broaden the scope of its applicability, and that of other interventions that aim at improving mental health care worldwide.

## Author Contributions

All authors contributed to the conceptual analyses. SP wrote the first draft of the manuscript. The remaining authors commented and modified successive drafts. All authors contributed and have approved the final manuscript.

## Conflict of Interest Statement

The authors declare that the research was conducted in the absence of any commercial or financial relationships that could be construed as a potential conflict of interest.
